# Conservative management of complete traumatic pancreatic body transection; A case report

**DOI:** 10.1016/j.ijscr.2020.05.011

**Published:** 2020-05-21

**Authors:** W. Duggan, E. Hannan, C. Brosnan, S. O'Sullivan, K. Conlon

**Affiliations:** St Vincent's University Hospital, 196 Merrion Road, Elm Park, Dublin, Ireland

## Abstract

**Introduction:**

Isolated pancreatic body transection secondary to blunt abdominal trauma is a very rare injury associated with poor outcomes. Almost all previously reported cases were managed by emergency distal pancreatectomy, which is associated with high morbidity and mortality. To our knowledge, this is the first reported case of complete transection of the pancreas at the body that was successfully treated by conservative management in an adult patient.

**Presentation of case:**

A 19-year-old male was found to have complete transection of the pancreatic body on computed tomography (CT) following blunt force abdominal trauma. Given that he was haemodynamically stable without evidence of haemorrhage or bile leakage on imaging, a trial of conservative management was initiated. He remained well through his admission, gradually improving clinically and biochemically with stable appearances on serial imaging. He remains asymptomatic as of six months since discharge from the hospital and continues to be monitored in the outpatient setting.

**Discussion:**

Management of pancreatic trauma with ductal injury has typically been with emergency distal pancreatectomy, which is associated with high morbidity and mortality. The decision to operate should not be purely based on radiological findings, and should take into account clinical status, haemodynamic stability, coexisting injuries and evidence of active haemorrhage or bile leak.

**Conclusion:**

In select cases, it is reasonable to trial conservative management in isolated traumatic pancreatic body fracture by means of close clinical observation and serial imaging. This may allow the patient to avoid a high-risk emergency distal pancreatectomy.

## Highlights

• Pancreatic trauma is associated with considerable morbidity and mortality.
• Traditionally, cases that involved injury to the pancreatic duct were almost always managed operatively.
• Much of the morbidity and mortality associated is related to operative complications.
• This case highlights that where possible, in instances where there is no bile leak or active haemorrhage, a conservative management approach may be safe and effective.

## 1 Introduction

Traumatic injury to the pancreas is uncommon, occurring in as few as 0.2% of blunt abdominal traumas [1]. Even rarer is isolated pancreatic injury, with almost all cases presenting with associated injuries [2]. Traumatic pancreatic fracture is associated with significant morbidity and mortality, with mortality rates as high as 34% [1]. Previously reported cases have almost all been managed by surgical intervention [3]. We report a case of a 19-year-old who was managed non-operatively following complete pancreatic transection as a result of blunt abdominal trauma. To our knowledge, this is the first reported case of complete transection of the pancreas at the body that was successfully treated by conservative management in an adult patient. This case has been reported in line with SCARE criteria [4].

## 2 Case Presentation

**Fig. 1:**
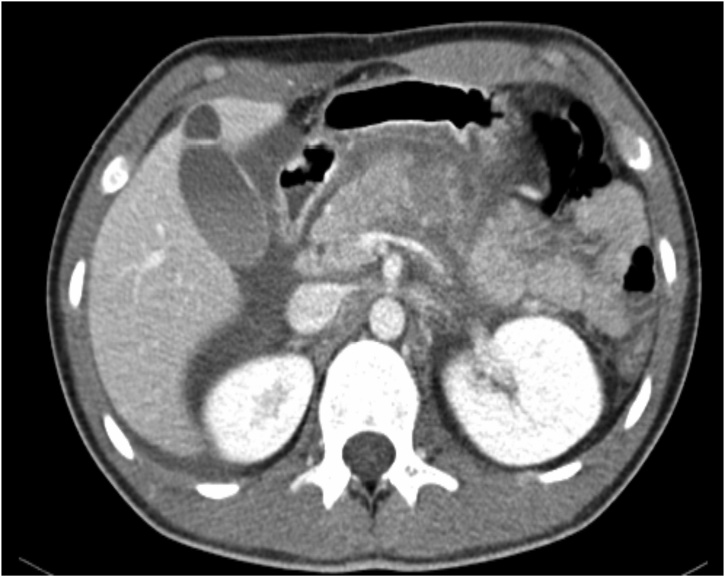
Axial view from CT abdomen & pelvis on admission - evidence of pancreatic body fracture with a pancreatic haematoma.

A 19-year-old male presented to the emergency department of a regional hospital with severe epigastric pain 12 hours after sustaining blunt trauma to the abdomen during an alleged assault. He was noted to be haemodynamically stable, and physical examination revealed epigastric tenderness and guarding. His white cell count (WCC) was 22.6 × 10^9^/L (normal range 4–9 × 10^9^/L) and his C-reactive protein (CRP) was 343 mg/L (normal range <3 mg/L). His amylase was also elevated at 378IU/L (normal range 25–125IU/L). His haemoglobin was within normal limits. An urgent computed tomogram (CT) was performed. This demonstrated complete transection of the pancreatic body with pancreatic haematoma formation (Fig. 1). This was consistent with a grade III injury according to the American Association for Surgery of Trauma (AAST) classification. No other injuries were identified and there was no evidence of active haemorrhage or bile leak.

The patient was promptly transferred to our centre where an on call hepatobiliary service was available. As the patient was haemodynamically stable with no evidence of active bleeding or bile leakage on his CT, he was treated conservatively. He was kept nil by mouth, placed on intravenous antibiotics and total parenteral nutrition was commenced via a peripherally inserted central catheter. Octreotide was also administered to reduce pancreatic secretion. He remained haemodynamically stable and gradually improved both clinically and biochemically. A pancreas protocol CT scan was performed on his fifth day in hospital, which showed stable appearances compared to the scan performed on his admission. The haematoma had remained stable, and once again there was no evidence of active bleeding or bile leak. Oral fluids were reintroduced at this point and intravenous antibiotics were stopped.

**Fig. 2:**
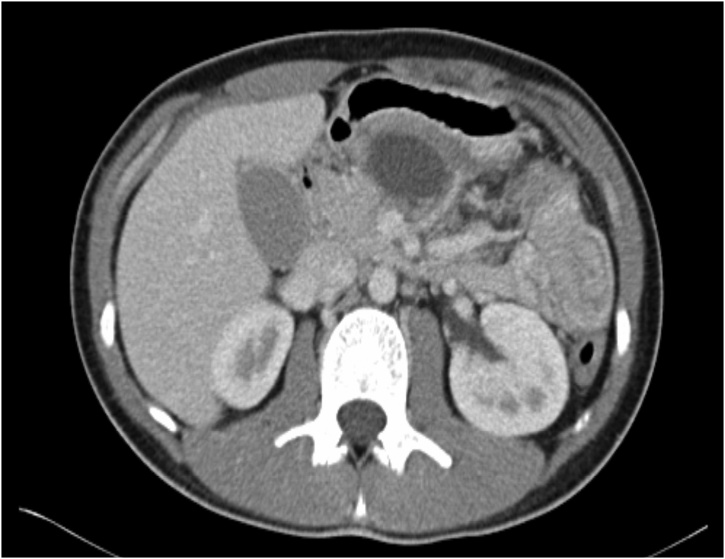
Follow up imaging on day 14 of admission demonstrating stable appearance of laceration with early pseudocyst formation.

A further CT scan was performed on day 14 which showed stable appearance of the laceration with maturation of the previously identified haematoma with adjacent pseudocyst formation (Fig. 2). At this point, the patient was completely asymptomatic, tolerating full diet, and both his WCC and CRP had normalised. He was discharged shortly after this and reviewed in outpatients 14 days following discharge with a further repeat CT scan. Once again, he remained asymptomatic and CT findings revealed minimal interval change. A magnetic resonance cholangiopancreatogram (MRCP) was subsequently carried out, confirming complete loss of pancreatic duct integrity. The duct was found to be dilated to 4.2 mm distal to the point of transection with associated hypo enhancement of the parenchyma suggestive of fibrosis. Despite this, the patient remains clinically well and is asymptomatic as of six months since discharge from the hospital. He continues to be monitored in the outpatient setting.

## 3 Discussion

Isolated pancreatic body transection is a rare but dangerous injury. The mechanism of injury is usually a result of direct compression of the pancreas against the lumbar vertebrae [1]. Children and slim young males appear to be particularly at risk due to the absence of the protective retroperitoneal fat pad [1], [2]. Pancreatic trauma is classified according to the AAST classification system [1]. Grade I and II injuries preserve ductal integrity, and are frequently managed by conservative measures [1]. Management for trauma where there is ductal injury has historically always been operative, with distal pancreatectomy frequently performed for grade III injuries [2]. However, emergency pancreatic resection in the context of trauma has been shown to have a high risk of morbidity (52.2%) and mortality (34.8%) [3]. Meneham et al demonstrated a mortality rate of surgically-managed pancreatic trauma as high as 27%, compared to a mortality rate associated with pancreatic trauma in general of 2%–17% [5].

We describe a case of a patient with an isolated AAST grade 3 pancreatic injury that has, to date, been successfully managed non-operatively, with a potentially very dangerous emergency laparotomy avoided. To our knowledge, this is the first such case in the literature in an adult patient. However, studies published in the paediatric population show that children with high grade pancreatic injuries may have better outcomes compared to operative management in regard to morbidity and mortality [6]. The decision to operate should not be purely based on the AAST radiological grading of trauma, but should instead be taken into account with a variety of other factors. This should include the presence of haemodynamic instability, evidence of active haemorrhage or bile leak on imaging, the presence of coexisting intra-abdominal injuries and the clinical status of the patient. In those where conservative management is deemed appropriate, the patient should be closely monitored for clinical and biochemical deterioration, with interval imaging to assess for changes that may necessitate surgical intervention. It is possible that our patient may go on to require surgical intervention in the future, and for this reason he continues to be monitored in the outpatient setting with follow-up imaging. However, if he does go on to require distal pancreatectomy, his initial conservative management will have allowed him to avoid a high risk emergency resection.

## 4 Conclusion

In select cases, isolated traumatic fracture of the body of the pancreas may be successfully managed conservatively, allowing the patient to avoid a potentially dangerous emergency distal pancreatectomy, which itself carries a high risk of morbidity and mortality. The decision to operate should not solely be based on the AAST radiological classification, but instead taken into account alongside the patient's clinical status, presence of haemodynamic instability, coexisting injuries or radiological evidence of active haemorrhage or bile leakage.

## Conflicts of interest

None to declare.

## Funding

No funding received.

## Ethical approval

This article is exempt from requiring ethical approval from our local ethics committee.

## Consent

Written informed consent for the publication of this case report was obtained from the patient.

## Author contributions

**WD:** Performed data acquisition, review of literature, drafting of original manuscript, final revisions.

**EH:** Drafting of original manuscript, final revisions.

**CB:** Drafting of original manuscript, final revisions.

**SO'S:** Data acquisition, review of literature.

**KC:** Expert advisory role, critical appraisal of article, final revision of manuscript.

## Registration of research studies

NA.

## Guarantor

William Duggan.

## Provenance and peer review

Not commissioned, externally peer-reviewed.
